# Mapping the zoonotic niche of Lassa fever in Africa

**DOI:** 10.1093/trstmh/trv047

**Published:** 2015-06-17

**Authors:** Adrian Q. N. Mylne, David M. Pigott, Joshua Longbottom, Freya Shearer, Kirsten A. Duda, Jane P. Messina, Daniel J. Weiss, Catherine L. Moyes, Nick Golding, Simon I. Hay

**Affiliations:** a Wellcome Trust Centre for Human Genetics, University of Oxford, Oxford, UK; b Department of Zoology, University of Oxford, Oxford, UK; c Institute for Health Metrics and Evaluation, University of Washington, Seattle, USA; d Fogarty International Center, National Institutes of Health, Bethesda, Maryland, USA

**Keywords:** Boosted regression trees, Lassa fever, LASV, *Mastomys natalensis*, Species distribution models, Viral haemorrhagic fever

## Abstract

**Background:**

Lassa fever is a viral haemorrhagic illness responsible for disease outbreaks across West Africa. It is a zoonosis, with the primary reservoir species identified as the Natal multimammate mouse, *Mastomys natalensis.* The host is distributed across sub-Saharan Africa while the virus' range appears to be restricted to West Africa. The majority of infections result from interactions between the animal reservoir and human populations, although secondary transmission between humans can occur, particularly in hospital settings.

**Methods:**

Using a species distribution model, the locations of confirmed human and animal infections with Lassa virus (LASV) were used to generate a probabilistic surface of zoonotic transmission potential across sub-Saharan Africa.

**Results:**

Our results predict that 37.7 million people in 14 countries, across much of West Africa, live in areas where conditions are suitable for zoonotic transmission of LASV. Four of these countries, where at-risk populations are predicted, have yet to report any cases of Lassa fever.

**Conclusions:**

These maps act as a spatial guide for future surveillance activities to better characterise the geographical distribution of the disease and understand the anthropological, virological and zoological interactions necessary for viral transmission. Combining this zoonotic niche map with detailed patient travel histories can aid differential diagnoses of febrile illnesses, enabling a more rapid response in providing care and reducing the risk of onward transmission.

## Introduction

In 1969, a previously undescribed disease with haemorrhagic symptoms was reported in two missionary nurses in the town of Lassa, Nigeria.^1^ The virus was subsequently identified as a novel member of the *Arenaviridae* family and named Lassa virus (*Lassa mammarenavirus*). Since then, this virus, which causes Lassa fever, has been reported in many West African countries with notable outbreaks in Guinea, Liberia, Nigeria and Sierra Leone. Evidence also indicates that the virus may have been present in West Africa long before the first detection date in 1969.^2^

The high seroprevelance for Lassa virus (LASV) specific antibodies in those Guinean (55%), Nigerian (21.3%) and Sierra Leonean (52%) populations tested indicates that most infections are mild or asymptomatic and do not require hospitalisation.^3–6^ This is supported by findings that more than 80% of persons who developed antibodies did not report a recent febrile illness.^3^ Overall mortality may be less than 5%, once mild or asymptomatic infections in the community are taken into account.^3^

**Figure 1.**
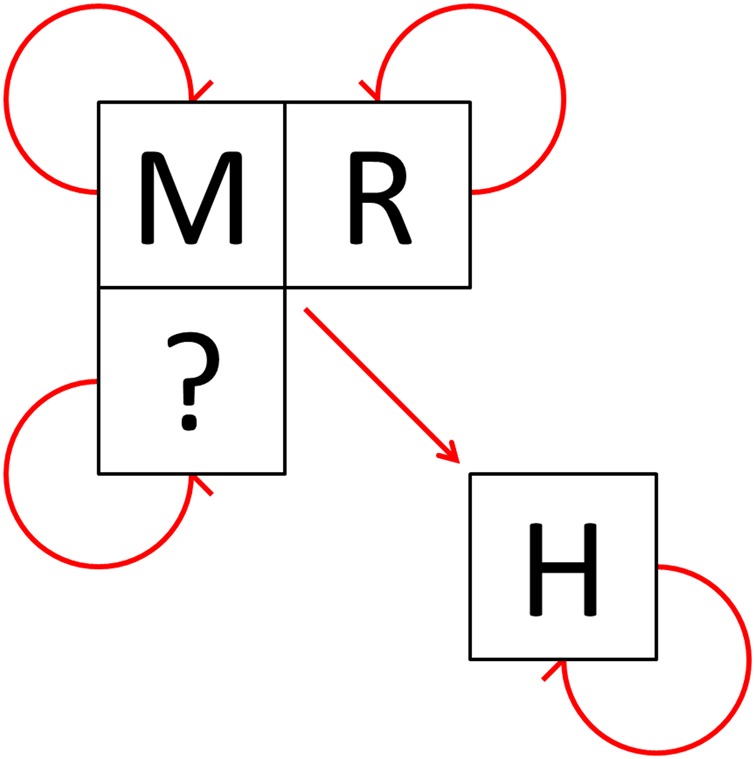
The epidemiology of Lassa virus transmission in West Africa.

LASV causes an acute viral haemorrhagic illness in a small fraction of those infected. The incubation period of Lassa fever ranges from 7–21 days with a wide range of clinical symptoms including headache, myalgia, fever, vascular bleeding and seizures as well as encephalopathy and oedema of the face and neck.^1,7–9^ Human-to-human transmission is possible through direct contact with infected blood or bodily fluid, although chains of transmission are often limited,^10–13^ especially if simple barrier nursing methods are implemented.^14–17^ A graphical representation of the epidemiology of Lassa virus transmission is presented in Figure 1.

‘M’ represents the suspected Natal multimammate mouse reservoir, *Mastomys natalensis*. ‘R’ represents other rodent species in which Lassa virus antibodies have been isolated including *Mastomys erythroleucus*, *Rattus rattus* and *Mus minutoides*.^3,18,19^ ‘H’ represents humans. The question mark indicates other potential species. Arrows indicate confirmed or suspected transmission cycles or infection routes. This figure is available in black and white in print and in color at Transactions online.

The low capacity for transmission between humans suggests a reservoir host is responsible for maintaining viral circulation in the environment. While LASV has been isolated from a number of rodent species, the majority of evidence implicates the Natal multimammate mouse, *Mastomys natalensis*, as the primary reservoir species.^3,18,19^ Seroprevalence has been reported to be as high as 60–80% in *M. natalensis* populations.^3,6^ Human infection can result from exposure to rodent excreta, hypothesised to be aerosolised, or through hunting/butchering of infected rodents for consumption.^3,20^

Recent studies^21^ suggest that outbreaks are largely fuelled by independent zoonotic transmission events from infected rodent hosts, whilst approximately 20% of cases result from secondary human-to-human transmission, typically through super-spreader events in hospital settings. This is in contrast to other blood-borne haemorrhagic viruses such as Ebola virus, for which human transmission chains are relatively long and fuel the majority of the outbreak.^22^

Lassa fever represents an importation risk across Africa and beyond,^23^ with a number of international cases reported.^13,24–28^ The ability of LASV to not only cause local outbreaks but also spread internationally provides a strong rationale for providing high-resolution mapping of Lassa fever risk across West Africa to aid differential diagnosis of viral haemorrhagic fevers.^23,29^ This paper aims to identify populations living in areas of environmental suitability for zoonotic transmission of LASV. We improve upon previous modelling efforts^30^ by including more recent outbreak data, animal infection records, and more refined environmental covariates.^31^ We also take advantage of recent advances in species distribution modelling techniques.^32,33^ This completes a series of papers that map the zoonotic niche of key viral haemorrhagic fevers, namely Ebola virus disease,^34,35^ Marburg virus disease^36^ and Crimean-Congo haemorrhagic fever,^37^ using comparable data collection and modelling methods. As a whole, this set of studies improves our understanding of the contemporary geographical distribution of these endemic and internationally important viral haemorrhagic fevers.^23,38^

## Materials and methods

### Methodological overview

A map defining areas of environmental suitability for zoonotic transmission of Lassa fever was generated using an ensemble boosted regression trees (BRT) species distribution modelling framework. BRT models combine large numbers of regression trees to model probability of species presence based on the values of environmental covariates.^39,40^ These models are trained using a spatial database of reported occurrences of infections in humans and animals alongside a set of background (or pseudo-absence) points representing environmental conditions in areas where cases are not reported. Areas that are environmentally similar to locations where zoonotic transmission of Lassa virus has been reported are thus identified. This approach requires a variety of information including: 1. reported index cases of Lassa fever; 2. Lassa virus detection in other mammalian hosts; 3. background (or pseudo-absence) information to represent locations where Lassa fever is likely absent; and 4. gridded surfaces of environmental covariates thought to influence Lassa fever distribution across Africa.

### Reported infections in humans and animal hosts

We supplemented existing Lassa fever datasets^30^ by searching for the terms ‘Lassa' on the literature search engines Web of Science, PubMed and Scopus. In addition, notifications of cases were obtained from ProMED,^41^ WHO and Public Health England. After initial assessment of abstracts for relevance, full text versions of articles thought likely to contain spatially explicit information on Lassa virus infection were obtained. When papers referred to articles with additional information not included in the original search, we also obtained these articles. In mapping the full extent of the zoonotic niche of Lassa fever, it is important to differentiate index cases from secondary cases resulting from human-to-human transmission. These two transmission routes should be considered as spatially distinct, with zoonotic transmission likely driven by environmental factors and secondary transmission by human behaviours and contact patterns. We therefore excluded records of infection if there was clear evidence that the case resulted from contact with another infected human (e.g., nosocomial transmission). Similarly, we excluded serosurveys of healthy individuals (due to the possibility of cross-reactivity with other viral agents and the uncertainty regarding time and place of infection) unless this could be linked to a prior fever retrospectively diagnosed as Lassa fever. If there was no indication of human-derived infection, the case was assumed to be of zoonotic origin. ProMED reports that described 'confirmed' cases were assumed to have been diagnosed using at least serological techniques.

Sites of infection were geo-positioned via Google Earth following a variation of existing protocols.^35^ The location for geo-positioning was either identified within the article, or assumed to have occurred within the vicinity of the individual's home. If the location was smaller than 5 km × 5 km in area, only the latitude and longitude for the site were recorded (termed a ‘point’). The remaining sites were treated as areas or ‘polygons’ and these were divided into three classes based on size. Areas of up to 10 km at their maximum width were defined by a circle of radius 5 km. Areas between 10 km and 25 km at their maximum width were defined as a circle of radius 12.5 km. The borders of larger areas were defined using either administrative division boundaries (as defined by the Global Administrative Units Layer, GAUL)^42^ or a bespoke polygon generated using geographic information system (GIS) software. Areas larger than one decimal degree squared were excluded. Following existing protocols, an occurrence of a case was defined as one or more cases of Lassa fever, within a specific geographical unit or 5 km × 5 km pixel in a given calendar year.^43,44^ In total, 104 articles were used to generate 203 points and 171 polygons. Of these: 62 data points were from the period 1969–1979; 13 from the period 1980–1989; 47 from the period 1990–1999; 171 from the period 2000–2009; 77 from the period 2010–2014 and 4 data points had no start or end date of infection.

Reports of infections in multimammate mice (*M. natalensis*) were confirmed either by serological or genetically based diagnostics. Geo-positioning was performed using the methodology outlined above.

### Covariates used in the analysis

A series of 5 km × 5 km gridded surfaces of a variety of environmental correlates thought to influence the distribution of Lassa fever were included as covariates in the model. These included information on the mean and range values for each pixel for land surface temperature (LST) (both night and day), enhanced vegetation indices (EVI), elevation and potential evapotranspiration (PET).^45,46^ For those surfaces derived from satellite imagery, gap-filling algorithms were used to correct anomalies caused by cloud cover.^31^ The gap-filled data span the years 2000 through 2015 and have a temporal resolution of 8 days. Data used in the models consist of mean values derived from all possible data points, resulting in synoptic surfaces that characterise normal conditions. As satellite data is widely unavailable for the years prior to 2000, we used summary raster values as indicators for the general long term environmental conditions. For additional information on environmental covariates see Weiss et al.^47^

Also included in the model was an estimated distribution of the primary reservoir host, the Natal multimammate mouse, *M. natalensis*. A separate species distribution model was carried out to capture the potential distribution of this rodent, using the same modelling procedure as for LASV (detailed below). Data for all members of the family Muridae were retrieved from the Global Biodiversity Information Facility (GBIF)^48^ totalling 2 228 003 records. From this, records of *M. natalensis* were identified and, prior to inclusion in the model, went through a quality control process whereby existing expert-opinion range maps,^49^ buffered by 100 km, were used to remove potentially erroneous results. In total 1031 occurrences were included in the analysis. Following existing approaches to deal with bias in observation and collection datasets,^50^ all other Muridae occurrences were used as background data, a required component for presence-background modelling approaches.^33^ Prior to inclusion in the final model, this prediction was clipped to within 500 km of the expert-opinion range map where the host species has been reported.

### Species distribution modelling framework

The occurrence datasets, combined with the covariate data outlined above, were then analysed using an ensemble boosted regression trees modelling framework.^40^ A total of 120 BRT submodels were run, with each iteration fitted to separate bootstrap-resampled datasets. Each resampled dataset had the same number of records as the full data, sampled randomly with replacement from the full dataset, subject to the constraint that at least five occurrence and five background records were present in each bootstrap. Model fitting was implemented using the gbm.step procedure in the dismo package in R.^51^ All tuning parameters of the algorithm were held at their default values (cross-validation folds = 10, tree complexity = 4, learning rate = 0.005, bag fraction = 0.75, step size = 10). In each run, the weighting of background data was adjusted so that the sum of weights of the background points equalled the weighted sum of the presence records to improve discrimination capacity of the model.^52^ A prediction map based upon the mean value for each 5 km × 5 km pixel across the 120 submodels was evaluated, as well as a 95% confidence interval around this value.

Accuracy of the models was analysed using the area under the curve (AUC) statistic. For each sub-model AUC was calculated as the mean of the cross-validated AUC across all 10-folds. The validation process divides the dataset into 10 groups of approximately equal presence and background data and assesses the ability of one subset to predict the remaining 90% of the data. These statistics were estimated using a pairwise distance sampling procedure in order to prevent inflation of the accuracy statistics due to spatial sorting bias.^53^ The overall mean and standard deviation of the ensemble AUC was then calculated from all the submodels.

### Modelling Lassa fever distribution

To incorporate spatial uncertainty in the location of outbreaks associated with polygon occurrence records, for each of the 120 submodels we randomly selected a different single point location within each occurrence polygon and treated this as the occurrence location. Since the final predictions were produced by averaging submodel predictions, this Monte Carlo procedure incorporated geographic uncertainty for some occurrence records in the final model.

These occurrence records of Lassa fever were then supplemented with 10 000 background points that were generated by randomly sampling across Africa, biased towards areas of higher population as a proxy for observation bias, under an assumption that more populous areas will be more likely to detect cases of Lassa fever should an outbreak occur.^34^

Previous investigations have indicated that accounting for differences in diagnostic accuracy can influence predictive ability^32^ and therefore several scenarios were considered. Iterations included altering the weighting between human or animal infections diagnosed via PCR and serological tests (ratios 1:1, 2:1, 4:1) as well as an iteration with only PCR diagnosed cases. Finally, a model was run using only human index cases.

### Evidence consensus and post-hoc masking

A variety of factors, not just environmental, influence the actual distribution of a species,^54–56^ some of which cannot be considered in the modelling framework due to a lack of data at the necessary resolution or an incomplete understanding of what drives the distribution. The evidence consensus framework provides a means by which areas modelled as environmentally suitable but do not have the disease due to, for instance, biogeographic reasons, can be masked out of the final analysis. The evidence consensus system takes information from a variety of sources, considering different aspects of Lassa fever epidemiology to characterise the consensus on the evidence for Lassa fever presence in a country.

Criteria considered included: 1. endemic status as defined by three health reporting organisations (WHO, CDC and the Global Infectious Diseases and Epidemiology Online Network); 2. reported infection in humans archived in peer-reviewed literature and other data sources, assessed for contemporariness and diagnostic accuracy; 3. outbreaks of Lassa fever characterised by size and contemporariness, where cases were absent, the likelihood of missed diagnosis was assessed by considering adjacency to countries with reported infection and healthcare expenditure as a proxy for diagnostic capacity and 4. animal infection information. A full methodology, including how these information sources relate, is outlined in detail in the Supplementary information S1. A threshold for risk was defined at −25 and was applied to Africa to define areas where Lassa fever presence is likely. This threshold was selected as it differentiated areas with high certainty of absence from regions where insufficient information was available to determine absence.

### Population living in areas of environmental suitability for Lassa virus transmission

A threshold probability for risk was determined by calculating the probability value that characterises 95% of all occurrences of Lassa fever (both human and animal data) turning the continuous environmental suitability surface into a binary at-risk, not-at-risk layer. Population sizes living in these 5 km × 5 km at-risk pixels were calculated from the WorldPop African population surface.^57,58^

## Results

### Reported infections in humans and animals

**Figure 2.**
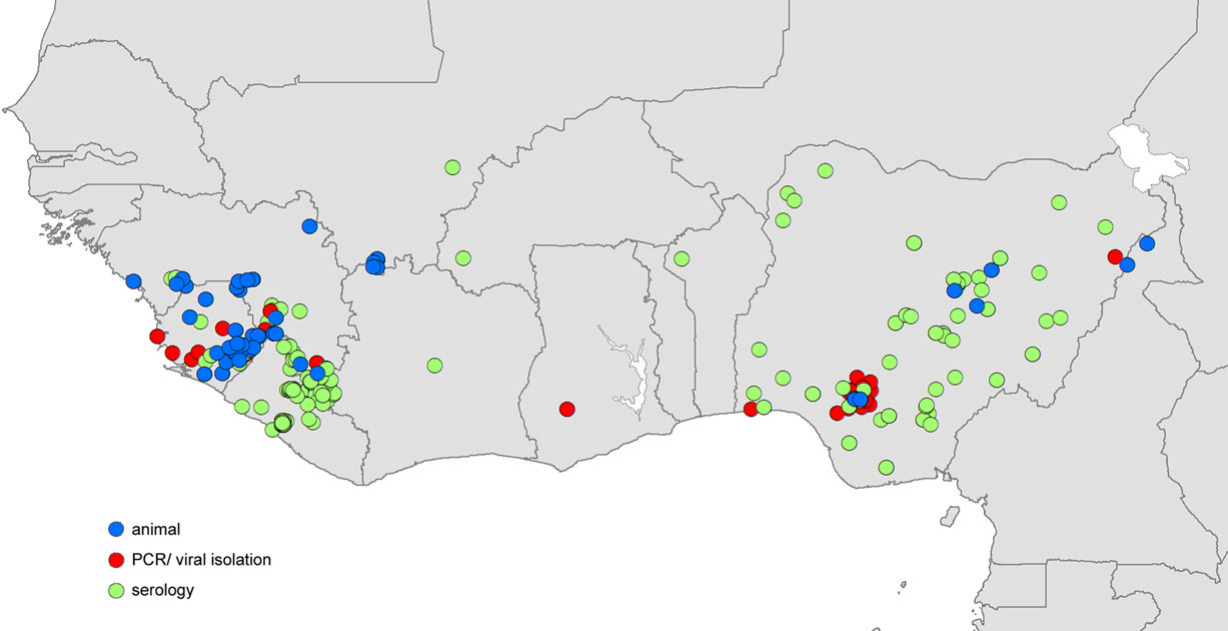
Reported locations of Lassa virus (LASV) infection used to build zoonotic niche maps.

In total, 374 distinct locations were identified as having animal infections or likely index cases of human outbreaks of Lassa fever (Figure 2). Human index cases were reported in nine different countries, mainly focussed in Liberia, Nigeria and Sierra Leone, but with some cases reported also in Benin, Burkina Faso, Côte d'Ivoire, Ghana, Guinea and Mali. Reports of infection in animals were found in four of these countries (Guinea, Mali, Nigeria and Sierra Leone) as well as in Cameroon, where no human index cases have been reported. The majority of human cases were diagnosed used serological techniques, although PCR diagnosis was often used in Nigeria and Sierra Leone (Table 1).

**Table 1.** Reported locations of Lassa virus infection used to build zoonotic niche maps

Country	Rodent data		Human data	
	PCR/viral isolate	Serology	PCR/viral isolate	Serology
Benin	0	0	0	1
Burkina Faso	0	0	0	1
Cameroon	0	2	0	0
Côte d'Ivoire	0	0	0	1
Ghana	0	0	2	0
Guinea	19	25	8	15
Liberia	0	0	2	69
Mali	8	0	0	1
Nigeria	3	6	49	88
Sierra Leone	12	15	20	27
TOTAL	42	48	81	203

Blue circles indicate location mid-points for animal LASV infection surveys. Red circles indicate location mid-points where human cases of Lassa fever were diagnosed using PCR or viral isolation methods. Green circles indicate location mid-points where human cases of Lassa fever were diagnosed using serological methods.

### Predicted rodent reservoir distribution

**Figure 3.**
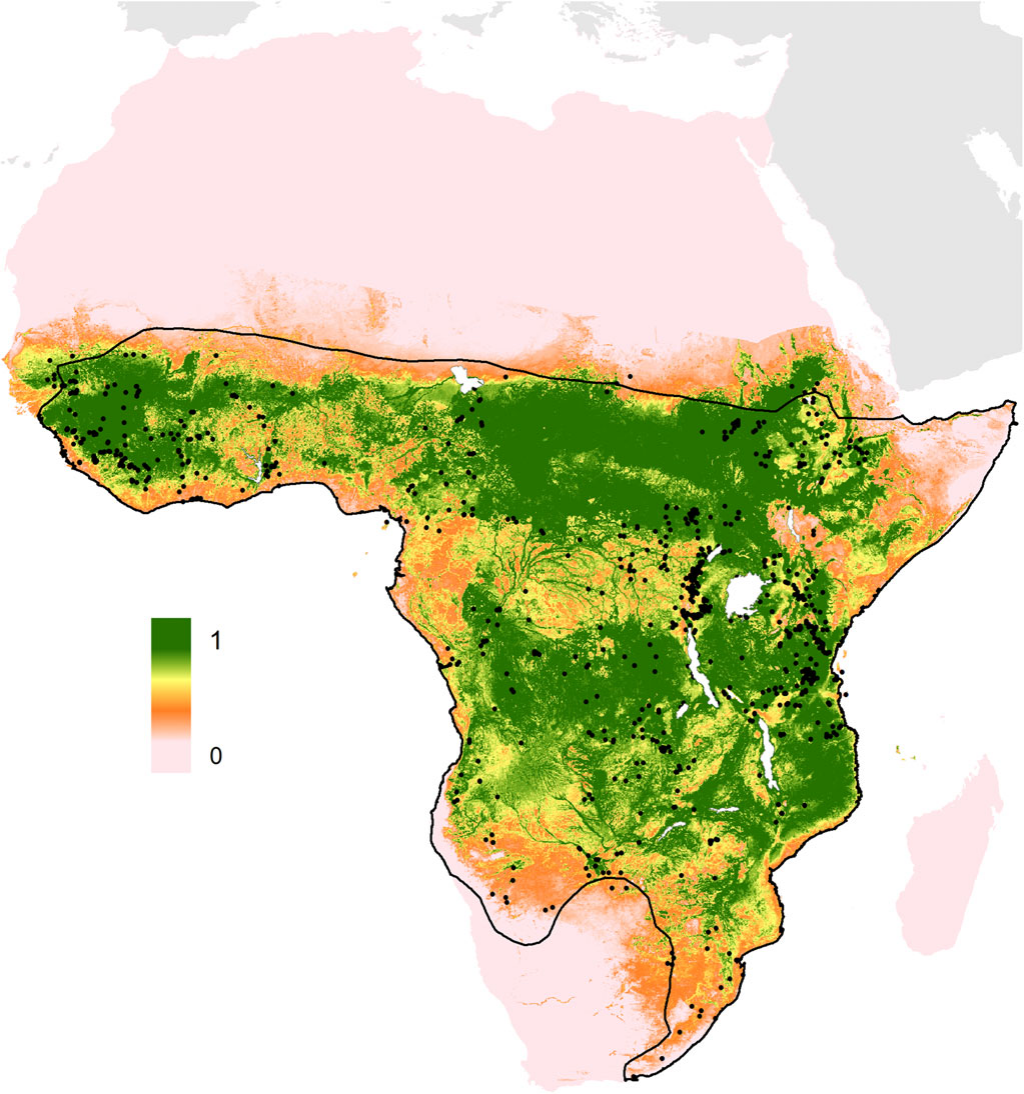
Predicted geographical distribution of the Natal multimammate mouse, *Mastomys natalensis*.

The Natal multimammate mouse, *M. natalensis,* was predicted to have a very broad distribution across sub-Saharan Africa ranging from West Africa, across to the horn of Africa, down to Natal province in eastern South Africa where it was first collected (Figure 3). The model identified vegetation indices (both EVI mean and range values), potential evapotranspiration, elevation (digital elevations models) and night time land surface temperature as the main predictors of environmental suitability for *M. natalensis* (Table 2). The AUC values were 0.63±0.01 indicating the model demonstrated moderate predictive skill.

**Table 2.** Summary statistics for model outputs. Relative contributions for each of the top five predictors are reported as a percentage

Statistic	Model 1*: Mastomys natalensis* distribution	Model 2: LASV zoonotic niche
AUC±standard deviation	0.63±0.01	0.79±0.02
1^st^ predictor	Mean EVI: 24.5%	Mean EVI: 26.5%
2^nd^ predictor	Mean PET: 19.7%	Night-time mean LST: 19.2%
3^rd^ predictor	Elevation (DEM): 16.3%	*M. natalensis* distribution: 13.6%
4^th^ predictor	Night-time mean LST: 14.8%	Elevation (DEM): 11.7%
5^th^ predictor	EVI range: 10.5%	Mean PET: 10.6%

AUC: area under the curve; DEM: digital elevations models; EVI: enhanced vegetation index; LASV: Lassa virus; LST: land surface temperature; PET: potential evapotranspiration.

The scale reflects the relative suitability of a given pixel for the presence of the Lassa virus zoonotic host, the Natal multimammate mouse, *Mastomys natalensis*. Areas closer to 1 (green) are more likely to harbour the rodent than those closer to 0 (pink). The prediction is clipped to within 500 km of the IUCN expert-opinion range map (solid black line),^49^ to remove environmentally suitable areas in which the mouse has never been reported. The black spots represent *M. natalensis* locations as reported by GBIF.

### Predictions for the zoonotic niche of Lassa virus

**Figure 4.**
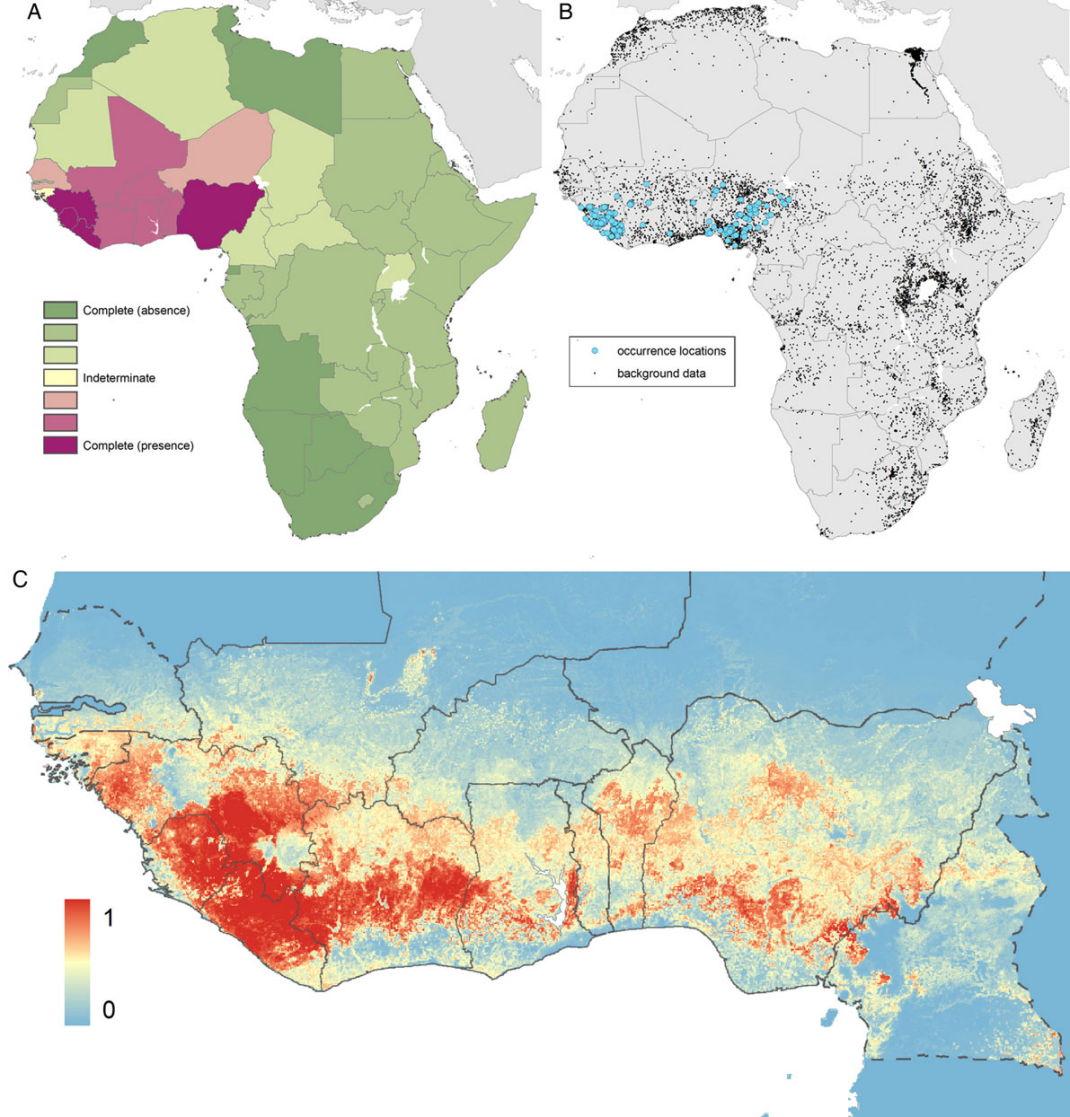
Maps of the: (A) definitive extents as determined by evidence consensus; (B) recorded occurrence and generated background pseudo-absence points used in the BRT procedure and (C) predicted geographical distribution of the zoonotic niche for Lassa virus.

The evidence consensus layer defined 13 countries as having consensus values ranging from complete consensus on presence to indeterminate. All countries reporting index cases of Lassa fever had a consensus score ranging from good to complete (above 60%). Togo had a moderate consensus on presence, while the remainder (Niger, Senegal, Guinea-Bissau and Cameroon) had either low consensus or indeterminate status (Figure 4).

Panel A shows the consensus on Lassa fever presence, ranging from dark green (complete consensus on absence) to purple (complete consensus on presence). Countries in yellow are those where evidence was inconclusive or contradictory for Lassa fever presence. Panel B shows the location of data points that went into the model. Panel C shows the environmental suitability for zoonotic transmission of Lassa virus. Areas closer to 1 (red) are more suitable than those closer to 0 (blue). Countries with borders outlined by a solid line are those where cases of LASV have previously been reported. Countries with borders outlined by a dash line have not previously reported LASV cases. The area under the curve statistic, calculated under a stringent cross-validation procedure is 0.79±0.02.

All model variants produced broadly consistent predictions across West Africa (see Supplementary information S2). Given these similarities, the equal weighting of occurrences based on diagnosis model was selected as this required the least assumptions and included the maximum amount of data. Large areas of environmental suitability for the zoonotic transmission of Lassa fever were found in Guinea, Liberia, Sierra Leone, Côte d'Ivoire and Nigeria, with smaller regions predicted in Benin, Burkina Faso, Togo, Mali, Senegal, Guinea-Bissau, Niger, Ghana and Cameroon (Figure 4).

The model identified vegetation, night-time land surface temperature, environmental suitability for the host species, elevation and potential evapotranspiration as the main predictors of suitability for zoonotic transmission of LASV (Table 2). The AUC value was 0.79±0.02 indicating the model demonstrated good predictive skill. Environmental covariate partial dependency plots are provided in the Supplementary information S3.

The full prediction surface without the evidence consensus mask is presented in the Supplementary information S2, where uncertainty maps for this prediction surface are also shown.

### Population at risk of zoonotic transmission of Lassa virus

The final threshold probability for risk, which captured 95% of all Lassa fever occurrences, was calculated to be equal to or greater than 0.646. In total, approximately 37.7 million individuals in 14 countries live in areas predicted to be environmentally suitable for the zoonotic transmission of LASV. The majority (97.9%) live in countries that have already reported index cases of Lassa fever, with Nigeria accounting for approximately 36% of the total population living in at-risk areas. More information is provided in the Supplementary information S4.

## Discussion

These maps present revised estimates of areas environmentally suitable for the zoonotic transmission of LASV and provide an important baseline for guiding Lassa fever surveillance and additional epidemiological investigations. Areas of environmental suitability are defined across a broad area of West Africa, including countries where no cases have been reported. These maps can therefore inform our wider understanding of the disease and aid future differential diagnosis of viral haemorrhagic fevers in areas where two or more viruses are potentially present.^23,38^

As with any model-based study, an awareness of data limitations and model assumptions is essential. Although predictive capability will be hindered by limited datasets where the true site of zoonotic transmission is unlikely to be reported, our study attempts to be as comprehensive as possible in including all known reports of zoonotic infections, as well as considering uncertainty in the location of initial infection. Because our models are only able to assess areas that are environmentally suitable for LASV, more research on how humans and animal reservoirs interact, as well as how the disease is transmitted within these populations, is needed to understand and translate this into true outbreak risk. Even with these limitations, we hope that our results will act as a springboard for further research to better understand the epidemiology of LASV and characterise the risk of this important VHF.

It is important to recognise that the outputs of this study are modelled estimates and are heavily influenced by the data used. Precisely geo-positioning the true site of infection is difficult to achieve. There is often an assumption that infection occurs in the locality of the patient's home address. Even when a patient's place of residence is documented, it may not represent the location where infection took place. Given the resolution of our zoonotic niche maps (5 km × 5 km), point locations cover a relatively broad area, which is likely to include the true infection site. For more uncertain locations, larger areas were defined to include the supposed site of infection and the model was adjusted accordingly.

In addition, the need for reliable information on the location of infection excluded hospital cases that did not document home location, because hospitals often serve a much broader area, particularly referral hospitals. Serosurveys of healthy individuals were also excluded, since seropositivity could reflect prior infection in a range of places and times. With rodent data, however, given their relatively limited dispersal ability,^59^ the assumption of infection occurring near trapping sites was valid.

Diagnostic accuracy was a potentially important consideration. We accounted for this factor by including a variety of weighting schema, as well as excluding potentially less reliable serological assays. This was also important given the spatial bias in the availability of specific diagnostic tests and, therefore, in the ability to accurately detect cases of Lassa fever. This spatial bias was reflected in the high proportion of cases seen in Nigeria and Sierra Leone where dedicated surveillance activity and research programmes exist.^60,61^ Using alternative diagnostic method weighting schemas to test model predictiveness, however, showed that this factor had little impact on the output maps and the validation and summary statistics.

These maps identify regions of environmental suitability for zoonotic transmission of LASV by defining a set of environmental conditions that best characterise the locations of known infections. Our model indicated mean EVI, night-time mean LST, *M. natalensis* suitability, elevation and PET as key predictors for environmental suitability. While inference of causal relationships cannot be directly evaluated using this approach, all these parameters have plausible influence on the transmission cycle of LASV and likely influence the rodent host population dynamics as well as the nature and frequency of human-rodent interactions. Indeed, other rodent-borne viruses, viral dynamics and corresponding disease risk have been shown to be strongly influenced by the environment.^62^ Further investigations must be undertaken to determine the precise nature of any causal relationship.

It is important to note, however, that while the environment has an important impact on influencing viral distribution, other factors will also impact upon its distribution.^54,63^ One means by which we accounted for this was by utilising the evidence consensus layer to assess the contemporary endemic status of a country from a variety of different sources. As a consequence, while large areas of sub-Saharan Africa are environmentally suitable for viral transmission, there is a consensus from other evidence,^64^ that the virus is not present. On the other hand, a number of countries (Guinea-Bissau, Senegal, Togo, Niger and Cameroon) are more likely to be underreporting cases due to their proximity to other endemic countries and their low healthcare expenditure. Whilst an imperfect process, the use of evidence consensus to define areas of likely under-reporting as opposed to regions of true absence is an important first step. Indeed, as Benin has recently proven, it is possible for outbreaks to occur in areas of previously reported absence.^65,66^

The results of this study act as a useful guide to help refine where potential at-risk populations exist and pinpoint where new surveys and surveillance initiatives would be most beneficial, particularly at the edges of the predicted geographical distribution of LASV (such as Cameroon and Senegal) and in countries where at-risk populations are predicted but have yet to report any cases of LASV (such as Togo). For more information regarding the predicted distribution of at-risk populations, see Supplementary information S4.

Areas suitable for disease transmission may, for other reasons, not result in cases. In addition, regions with similar environments may report very different caseloads. Therefore, translating these environmental suitability layers into actual incidence and prevalence estimates requires more detailed epidemiological information regarding the interactions between virus, host and human, and how the three relate across different spatial scales. The maps presented here act as a baseline for further refinement when additional spatial information becomes available.^67^ Evidence is most needed in areas where the host is found but cases have not been reported. For instance, while the primary reservoir host is found across Africa, the virus itself appears to be restricted to West Africa. Could this be related to the seemingly poor dispersal ability of the host, or are there biogeographic barriers preventing mixing of the populations? For example, with bat-borne viruses, the likelihood of infection in bat colonies on the fringes of their distribution is greater than found here for LASV in mice, likely due to the superior dispersal ability of bats.^68,69^ Understanding the viral dynamics within the reservoir host, and how reservoir populations spread the virus between themselves, may provide the critical step in understanding true risk. It is also unknown whether all human populations are equally susceptible to infection. Anthropological studies suggest that different groups behave very differently with regards to mammal species and subtle variations in housing conditions, social relations and agricultural practices could have large impacts on how humans and disease reservoirs interact.^70^

### Conclusions

The output maps provide an important resource for refining our understanding of the distribution of Lassa fever. Baseline estimates such as these are necessary, not only to aid in selecting locations for initial surveys in areas beyond those that currently report cases and directing both human and animal surveillance activities, but also to inform a wider community of public health officials about the potential risk of Lassa fever. Given the potential for nosocomial transmission of this disease,^10,11,71^ and especially given the potential for misdiagnosis as other febrile illnesses,^72,73^ incorporating Lassa fever in a differential diagnosis is critical for timely prevention measures to be put in place. In an increasingly connected world, these maps not only inform local risk, but can assist in detection of potential imported cases when a travel history is available. As the recent Ebola virus disease outbreak in West Africa has shown, recognising this risk can be a vital first step in preventing further transmission of this important viral haemorrhagic fever.

## Supplementary data

Supplementary data are available at Transactions online (http://trstmh.oxfordjournals.org/).

Supplementary Data
