# Supplementary material for: Mapping the zoonotic niche of Lassa fever in Africa
Source: Trans R Soc Trop Med Hyg. 2015 Jun 17;109(8):483–92. doi: 10.1093/trstmh/trv047 (PMC4501400; doi:10.1093/trstmh/trv047)
Supplement: Supplementary Data [file supp_109_8_483__index.html]

Mapping the zoonotic niche of Lassa fever in Africa — Supplementary Data 

# Mapping the zoonotic niche of Lassa fever in Africa

## Supplementary Data

Supplementary Data

- Supplementary Information 1 - docx file
- Supplementary Information 2 - docx file
- Supplementary Information 3 - docx file
- Supplementary Information 4 - docx file
