# Supplementary material for: Mapping the zoonotic niche of Lassa fever in Africa
Source: Trans R Soc Trop Med Hyg. 2015 Jun 17;109(8):483–92. doi: 10.1093/trstmh/trv047 (PMC4501400; doi:10.1093/trstmh/trv047)
Supplement: Supplementary Data [file supp_trv047_trv047supp_supplementaryinformation1.docx]

**Supplementary information S1: evidence consensus**

**Methodology**

Evidence consensus is designed to assess a variety of information sources to evaluate the likely presence or absence of a disease in a specific country.[^1^](#_ENREF_1) Whilst highlighting countries where conflicting information or data gaps and uncertainty cloud the ability for a definitive presence/absence assessment is important in its own right, these surfaces can also be incorporated into models of disease risk as a way of informing background data selection and masking out areas which are environmentally suitable for transmission of disease, but where the disease is absent for other reasons such as human control or biogeographic features.[^2-4^](#_ENREF_2) Four main data sources were used to assess the variety of information associated with Lassa fever across the African continent. They included: health organisation status; peer-reviewed literature evidence of infection in humans; case data looking at outbreak sizes and animal infection information.

**Health organisation status (-3 to +3)**

Three bodies that assess national status of endemicity for Lassa fever were included: WHO,[^5^](#_ENREF_5) CDC[^6^](#_ENREF_6) and the Global Infectious Diseases and Epidemiology Online Network (GIDEON).[^7^](#_ENREF_7) For each organisation, +1 was scored if a country was indicated as disease endemic or had the disease present and -1 was scored if Lassa fever was reported as absent.

**Reports of human infection (+2 to +6)**

A search for articles on Lassa fever was performed using PubMed, Web of Science and Scopus. Within these articles, cases of Lassa fever in humans were identified for as many African countries as possible. Each case was evaluated for contemporariness (+3 2007-2014, +2 1999-2006, +1 1998 and earlier) and diagnostic accuracy (+3 for PCR or genetic isolation, +2 for serological based detection, +1 for reported cases without diagnostic support). The highest scoring report was included in the final table.

**Case data for outbreaks (-6 to +6)**

Outbreaks were identified and assessed by size and contemporariness as outlined below:

| Case numbers | Date | Score |
| --- | --- | --- |
| 20+ | 2007-2014 | +6 |
| 20+ | 1999-2006 | +5 |
| 20+ | 1998 and earlier | +4 |
| 5-19 | 2007-2014 | +3 |
| 5-19 | 1999-2006 | +2 |
| 5-19 | 1998 and earlier | +1 |

Where no outbreak was identified, we assessed healthcare expenditure and adjacency to countries with reported cases to quantify the likelihood of genuine absence versus underreporting. Information on healthcare expenditure was derived from the WHO World Health Statistics [^8^](#_ENREF_8) listing of per capita total expenditure on health at the average exchange rate in USD for the year 2011. Healthcare expenditure was categorised into three classes: Healthcare Expenditure (HE) Low (<$150), HE Medium ($150<x<$500), HE High (>$500). Adjacency was considered where countries shared a common border. Adjacency was categorised into four classes: 1. no neighbours reported cases of Lassa fever; 2. one neighbour reported cases (unless the country only bordered one or two countries); 3. two neighbours reported cases (unless the country only bordered two countries) or half of the bordering countries had cases and 4. three neighbours reported cases or all neighbouring countries had cases. The complication in categorisation was necessary in order to avoid unfairly penalising those countries with fewer shared borders. Healthcare expenditure and adjacency were then considered within the context of each other and scores assigned as follows:

| Healthcare class | No neighbours | One neighbour | Two neighbours/half of all neighbours | Three or more neighbours/all neighbours |
| --- | --- | --- | --- | --- |
| HE Low | -2 | 0 | +3 | +6 |
| HE Medium | -4 | 0 | +2 | +5 |
| HE High | -6 | 0 | +1 | +4 |

**Animal infection (+1)**

In addition to collecting data on human infections, animal infection reports were also included. If there was evidence of infection in the rodent host (the Natal multimammate mouse, *Mastomys natalensis*) +1 was added as a supplementary score.

**Final evaluation**

All the scores were added together to generate the final evidence consensus value. The denominator (maximum possible score) varied depending on which criteria were included. If no peer-review literature cases could be found the denominator was 9 (3 from Health Reporting Organisation status and 6 from case data/healthcare expenditure status). If peer-reviewed reports were present, the denominator was 15 (3 from Health Reporting Organisation status, 6 from the peer-review literature cases and 6 from case data/healthcare expenditure status). Where a country had evidence of rodent infection, the denominator was increased by 1 (i.e., /10 or /16). The final score was then reported as a percentage. The full evidence consensus table is outlined below.

**Table S1.1. Evidence consensus**

| Country | GIDEON | WHO | CDC | Peer Review | Case Data | Animal | Score |
| --- | --- | --- | --- | --- | --- | --- | --- |
| Algeria | Not endemic | Absent | Absent  -3 | - | Neighbours Mali  HE Medium ($233)  0 | - | -3+0/9  -33% |
| Angola | Not endemic | Absent | Absent  -3 | - | No neighbours  HE Medium ($178)  -4 | - | -3-4/9  -78% |
| Benin | Not endemic | Present | Present  +1 | 2014  Serology [^9^](#_ENREF_9)  +5 | 15 cases in 2014 [^9^](#_ENREF_9)  +3 | - | 1+5+3/15  +60% |
| Botswana | Not endemic | Absent | Absent  -3 | - | No neighbours  HE Medium ($404)  -4 | - | -3-4/9  -78% |
| Burkina Faso | Endemic | Absent | Present  +1 | 1975  Serology [^10^](#_ENREF_10)  +3 | Neighbours Ivory Coast, Ghana, Benin, and Mali  HE Low ($39)  +6 | - | 1+3+6/15  +67% |
| Burundi | Not endemic | Absent | Absent  -3 | - | No neighbours  HE Low ($21)  -2 | - | -3-2/9  -56% |
| Cameroon | Not endemic | Absent | Absent  -3 | - | Neighbours Nigeria  HE Low ($64)  0 | Serological evidence in 1989 [^11^](#_ENREF_11)  +1 | -3+0+1/10  -20% |
| Cape Verde | Not endemic | Absent | Absent  -3 | - | No neighbours  HE Medium ($153)  -4 | - | -3-4/9  -78% |
| Central African Republic | Endemic | Absent | Absent  -1 | - | No neighbours  HE Low ($19)  -2 | - | -1-2/9  -33% |
| Chad | Not endemic | Absent | Absent  -3 | - | Neighbours Nigeria  HE Low ($25)  0 | - | -3+0/9  -33% |
| Comoros | Not endemic | Absent | Absent  -3 | - | No neighbours  HE Low ($31)  -2 | - | -3-2/9  -56% |
| Congo | Not endemic | Absent | Absent  -3 | - | No neighbours  HE Low ($85)  -2 | - | -3-2/9  -56% |
| Cote d'Ivoire | Endemic | Absent | Present  +1 | 1975  Serology [^10^](#_ENREF_10)  +3 | All neighbours have reported Lassa cases  HE Low ($84)  +6 | - | 1+3+6/15  +67% |
| Democratic Republic of the Congo | Not endemic | Absent | Absent  -3 | - | No neighbours  HE Low ($15)  -2 | - | -3-2/9  -56% |
| Djibouti | Not endemic | Absent | Absent  -3 | - | No neighbours  HE Low ($119)  -2 | - | -3-2/9  -56% |
| Egypt | Not endemic | Absent | Absent  -3 | - | No neighbours  HE Low ($137)  -2 | - | -3-2/9  -56% |
| Equatorial Guinea | Not endemic | Absent | Absent  -3 | - | No neighbours  HE High ($1051)  -6 | - | -3-6/9  -100% |
| Eritrea | Not endemic | Absent | Absent  -3 | - | No neighbours  HE Low ($12)  -2 | - | -3-2/9  -56% |
| Ethiopia | Not endemic | Absent | Absent  -3 | - | No neighbours  HE Low ($14)  -2 | - | -3-2/9  -56% |
| Gabon | Endemic | Absent | Absent  -1 | - | No neighbours  HE Medium ($401)  -4 | - | -1-4/9  -56% |
| Gambia | Not endemic | Absent | Absent  -3 | - | No neighbours  HE Low ($24)  -2 | - | -3-2/9  -56% |
| Ghana | Endemic | Absent | Present  +1 | 2012  PCR [^12^](#_ENREF_12)  +6 | Neighbours Ivory Coast and Burkina Faso  HE Low ($83)  +3 | - | 1+6+3/15  +67% |
| Guinea | Endemic | Present | Present  +3 | 2013  PCR [^13^](#_ENREF_13)  +6 | 22 cases between 1996 and 1999 [^14^](#_ENREF_14)  +2 | Multiple infections reported [^15^](#_ENREF_15)^,^ [^16^](#_ENREF_16)  +1 | 3+6+2+1/16  +75% |
| Guinea-Bissau | Not endemic | Absent | Absent  -3 | - | Neighbours Guinea  HE Low ($35)  +3 | - | -3+3/9  0% |
| Kenya | Not endemic | Absent | Absent  -3 | - | No neighbours  HE Low ($35)  -2 | - | -3-2/9  -56% |
| Lesotho | Not endemic | Absent | Absent  -3 | - | No neighbours  HE Low ($35)  -2 | - | -3-2/9  -56% |
| Liberia | Endemic | Present | Present  +3 | 2010  PCR [^17^](#_ENREF_17)  +6 | 21 cases in 2007 [^18-20^](#_ENREF_18)  +6 | - | 3+6+6/15  +100% |
| Libya | Not endemic | Absent | Absent  -3 | - | No neighbours  HE Medium ($211)  -4 | - | -3-4/9  -78% |
| Madagascar | Not endemic | Absent | Absent  -3 | - | No neighbours  HE Low ($19)  -2 | - | -3-2/9  -56% |
| Malawi | Not endemic | Absent | Absent  -3 | - | No neighbours  HE Low ($30)  -2 | - | -3-2/9  -56% |
| Mali | Endemic | Absent | Present  +1 | 1975  Serology [^10^](#_ENREF_10)  +3 | Neighbours Guinea, Ivory Coast and Burkina Faso  HE Low ($51)  +6 | PCR isolation in 2013 [^21^](#_ENREF_21)  +1 | 1+3+6+1/16  +69% |
| Mauritania | Not endemic | Absent | Absent  -3 | - | Neighbours Mali  HE Low ($51)  0 | - | -3+0/9  -33% |
| Mauritius | Not endemic | Absent | Absent  -3 | - | No neighbours  HE Medium ($450)  -4 | - | -3-4/9  -78% |
| Mayotte | Not endemic | Absent | Absent  -3 | - | No neighbours  Assumed HE High  -6 | - | -3-6/9  -100% |
| Morocco | Not endemic | Absent | Absent  -3 | - | No neighbours  HE Medium ($195)  -4 | - | -3-4/9  -78% |
| Mozambique | Not endemic | Absent | Absent  -3 | - | No neighbours  HE Low ($33)  -2 | - | -3-2/9  -56% |
| Namibia | Not endemic | Absent | Absent  -3 | - | No neighbours  HE Medium ($486)  -4 | - | -3-4/9  -78% |
| Niger | Not endemic | Absent | Absent  -3 | - | Neighbours Nigeria, Benin, Burkina Faso and Mali  HE Low ($25)  +6 | - | -3+6/9  +33% |
| Nigeria | Endemic | Present | Present  +3 | 2013  PCR [^22^](#_ENREF_22)^,^ [^23^](#_ENREF_23)  +6 | 208 cases in 2014 [^24^](#_ENREF_24)  +6 | Serological evidence (2005) [^25^](#_ENREF_25) and viral isolation (1975) [^26^](#_ENREF_26)  +1 | 3+6+6+1/16  +100% |
| Réunion | Not endemic | Absent | Absent  -3 | - | No neighbours  Assumed HE High  -6 | - | -3-6/9  -100% |
| Rwanda | Not endemic | Absent | Absent  -3 | - | No neighbours  HE Low ($62)  -2 | - | -3-2/9  -56% |
| Sao Tome and Principe | Not endemic | Absent | Absent  -3 | - | No neighbours  HE Low ($108)  -2 | - | -3-2/9  -56% |
| Senegal | Endemic | Absent | Absent  -1 | - | Neighbours Guinea and Mali  HE Low ($54)  +3 | - | -1+3/9  +22% |
| Seychelles | Not endemic | Absent | Absent  -3 | - | No neighbours  HE Medium ($413)  -4 | - | -3-4/9  -78% |
| Sierra Leone | Endemic | Present | Present  +3 | 2011  PCR [^27^](#_ENREF_27)  +6 | 153 cases in 2010 [^28^](#_ENREF_28)  +6 | Multiple infections reported [^29^](#_ENREF_29)^,^ [^30^](#_ENREF_30)  +1 | 3+6+6+1/16  +100% |
| Somalia | Not endemic | Absent | Absent  -3 | - | No neighbours  Assumed HE Low  -2 | - | -3-2/9  -56% |
| South Africa | Not endemic | Absent | Absent  -3 | - | No neighbours  HE High ($670)  -6 | - | -3-6/9  -100% |
| South Sudan | Not endemic | Absent | Absent  -3 | - | No neighbours  HE Low ($32)  -2 | - | -3-2/9  -56% |
| Sudan | Not endemic | Absent | Absent  -3 | - | No neighbours  HE Low ($119)  -2 | - | -3-2/9  -56% |
| Swaziland | Not endemic | Absent | Absent  -3 | - | No neighbours  HE Medium ($270)  -4 | - | -3-4/9  -78% |
| Togo | Not endemic | Absent | Present  -1 | - | All neighbours have reported cases  HE Low ($43)  +6 | - | -1+6/9  +56% |
| Tunisia | Not endemic | Absent | Absent  -3 | - | No neighbours  HE Medium ($304)  -4 | - | -3-4/9  -77% |
| Uganda | Endemic | Absent | Absent  -1 | - | No neighbours  HE Low ($41)  -2 | - | -1-2/9  -33% |
| United Republic of Tanzania | Not endemic | Absent | Absent  -3 | - | No neighbours  HE Low ($38)  -2 | - | -3-2/9  -56% |
| Western Sahara | Not endemic | Absent | Absent  -3 | - | No neighbours  Assumed HE Low  -2 | - | -3-2/9  -56% |
| Zambia | Not endemic | Absent | Absent  -3 | - | No neighbours  HE Low ($87)  -2 | - | -3-2/9  -56% |
| Zimbabwe | Not endemic | Absent | Absent  -3 | - | No neighbours  Assumed HE Low  -2 | - | -3-2/9  -56% |


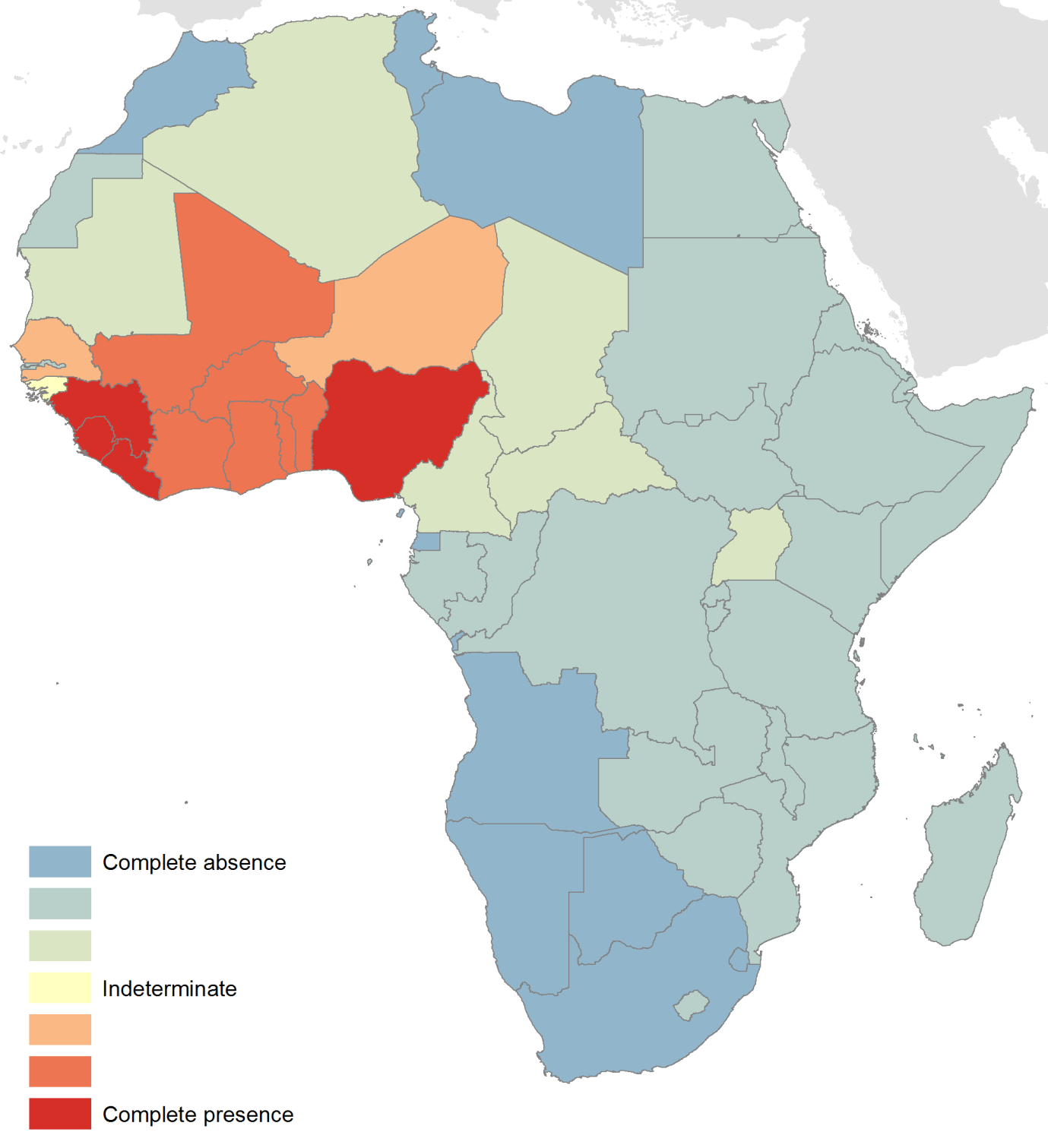


**Figure S1.1. Map of Africa showing evidence consensus scores for Lassa fever presence/absence.** Areas in red indicate consensus on disease presence whilst areas in blue have consensus on disease absence.

**References**

1. Brady OJ, Gething PW, Bhatt S et al. Refining the global spatial limits of dengue virus transmission by evidence-based consensus. PLoS Negl Trop Dis 2012;6:e1760.

2. Pigott DM, Bhatt S, Golding N et al. Global distribution maps of the leishmaniases. elife 2014;3:e02851.

3. Bhatt S, Gething PW, Brady OJ et al. The global distribution and burden of dengue. Nature 2013;496:504-7.

4. Cano J, Rebollo MP, Golding N et al. The global distribution and transmission limits of lymphatic filariasis: past and present. Parasit Vectors 2014;7:466.

5. WHO. Lassa fever - fact sheet #179. Geneva: World Health Organization; 2015. <http://www.who.int/mediacentre/factsheets/fs179/en/> [accessed 25 March 2015].

6. CDC. Lassa fever. <http://www.cdc.gov/vhf/lassa/> [accessed 25 March 2015].

7. Edberg SC. Global Infectious Diseases and Epidemiology Network (GIDEON): a world wide Web-based program for diagnosis and informatics in infectious diseases. Clin Infect Dis 2005;40:123-6.

8. WHO. World Health Statistics 2014. Geneva: World Health Organization; 2014.

9. ProMED-mail. Lassa fever - Benin (02) 20141126.2992727. [www.promedmail.org](http://www.promedmail.org) [accessed 24 March 2015].

10. Frame JD. Surveillance of Lassa fever in missionaries stationed in West Africa. Bull World Health Organ 1975;52:593-8.

11. Gonzalez JP, Josse R, Johnson ED et al. Antibody prevalence against haemorrhagic fever viruses in randomized representative Central African populations. Res Virol 1989;140:319-31.

12. Dzotsi EK, Ohene SA, Asiedu-Bekoe F et al. The first cases of Lassa fever in Ghana. Ghana Med J 2012;46:166-70.

13. Klempa B, Koulemou K, Auste B et al. Seroepidemiological study reveals regional co-occurrence of Lassa- and Hantavirus antibodies in Upper Guinea, West Africa. Trop Med Int Health 2013;18:366-71.

14. Bausch DG, Demby AH, Coulibaly M et al. Lassa fever in Guinea: I. Epidemiology of human disease and clinical observations. Vector Borne Zoonotic Dis 2001;1:269-81.

15. Lalis A, Leblois R, Lecompte E et al. The impact of human conflict on the genetics of Mastomys natalensis and Lassa virus in West Africa. PLoS One 2012;7:e37068.

16. Fichet-Calvet E, Lecompte E, Koivogui L et al. Fluctuation of abundance and Lassa virus prevalence in Mastomys natalensis in Guinea, West Africa. Vector Borne Zoonotic Dis 2007;7:119-28.

17. Amorosa V, MacNeil A, McConnell R et al. Imported Lassa fever, Pennsylvania, USA, 2010. Emerg Infect Dis 2010;16:1598-600.

18. ProMED-mail. Lassa fever - Liberia: RFI 20070410.1210. [www.promedmail.org](http://www.promedmail.org) [accessed 24 March 2015].

19. ProMED-mail. Lassa fever - Liberia (02): confirmed 20070413.1235. [www.promedmail.org](http://www.promedmail.org) [accessed 24 March 2015].

20. ProMED-mail. Lassa fever - Liberia (03) 20070430.1406. [www.promedmail.org](http://www.promedmail.org) [accessed 24 March 2015].

21. Safronetz D, Sogoba N, Lopez JE et al. Geographic distribution and genetic characterization of Lassa virus in sub-Saharan Mali. PLoS Negl Trop Dis 2013;7:e2582.

22. Dongo AE, Kesieme EB, Iyamu CE et al. Lassa fever presenting as acute abdomen: a case series. Virol J 2013;10:123.

23. Ajayi NA, Nwigwe CG, Azuogu BN et al. Containing a Lassa fever epidemic in a resource-limited setting: outbreak description and lessons learned from Abakaliki, Nigeria (January-March 2012). Int J Infect Dis 2013;17:e1011-6.

24. ProMED-mail. Lassa fever - Nigeria, Liberia 20140328.2363217. [www.promedmail.org](http://www.promedmail.org) [accessed 24 March 2015].

25. Okoror LE, Esumeh FI, Agbonlahor DE et al. Lassa virus: seroepidemiological survey of rodents caught in Ekpoma and environs. Trop Doct 2005;35:16-7.

26. Wulff H, Fabiyi A, Monath TP. Recent isolations of Lassa virus from Nigerian rodents. Bull World Health Organ 1975;52:609-13.

27. Branco LM, Boisen ML, Andersen KG et al. Lassa hemorrhagic fever in a late term pregnancy from northern Sierra Leone with a positive maternal outcome: case report. Virol J 2011;8:404.

28. ProMED-mail. Lassa fever - Sierra Leone (04): (NO) 20101111.4101. [www.promedmail.org](http://www.promedmail.org) [accessed 24 March 2015].

29. Leski TA, Stockelman MG, Moses LM et al. Sequence variability and geographic distribution of lassa virus, Sierra Leone. Emerg Infect Dis 2015;21:609-18.

30. McCormick JB, Webb PA, Krebs JW et al. A prospective study of the epidemiology and ecology of Lassa fever. J Infect Dis 1987;155:437-44.
