# Supplementary material for: Mapping the zoonotic niche of Lassa fever in Africa
Source: Trans R Soc Trop Med Hyg. 2015 Jun 17;109(8):483–92. doi: 10.1093/trstmh/trv047 (PMC4501400; doi:10.1093/trstmh/trv047)
Supplement: Supplementary Data [file supp_trv047_trv047supp_supplementaryinformation2.docx]

**Supplementary Information S2: model outputs**

**Figure S2.1. Predicted geographical distribution of the zoonotic niche for Lassa virus using a 1:1 ratio diagnostic weighting schema** **for human or animal infections diagnosed via PCR/viral isolation and serological tests, respectively (Model 1).**

The scale reflects the environmental suitability for zoonotic transmission of Lassa virus. Areas closer to 1 (red) are more suitable than those closer to 0 (blue). The area under the curve statistic, calculated under a stringent cross-validation procedure is 0.79±0.02.


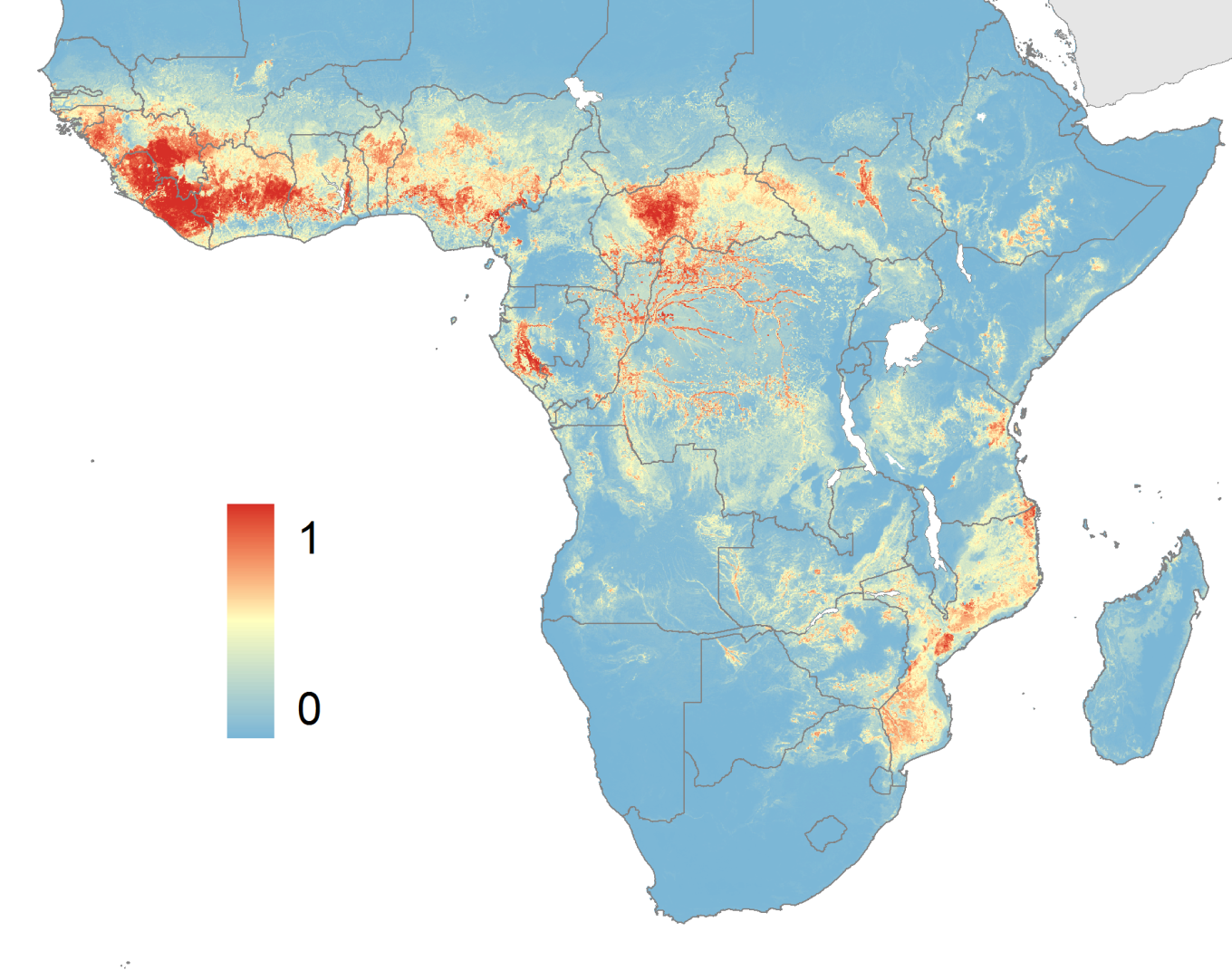


**Figure S2.2. Predicted geographical distribution of the zoonotic niche for Lassa virus using a 2:1 ratio diagnostic weighting schema** **for human or animal infections diagnosed via PCR/viral isolation and serological tests, respectively (Model 2).**

The scale reflects the environmental suitability for zoonotic transmission of Lassa virus. Areas closer to 1 (red) are more suitable than those closer to 0 (blue). The area under the curve statistic, calculated under a stringent cross-validation procedure is 0.79±0.02.


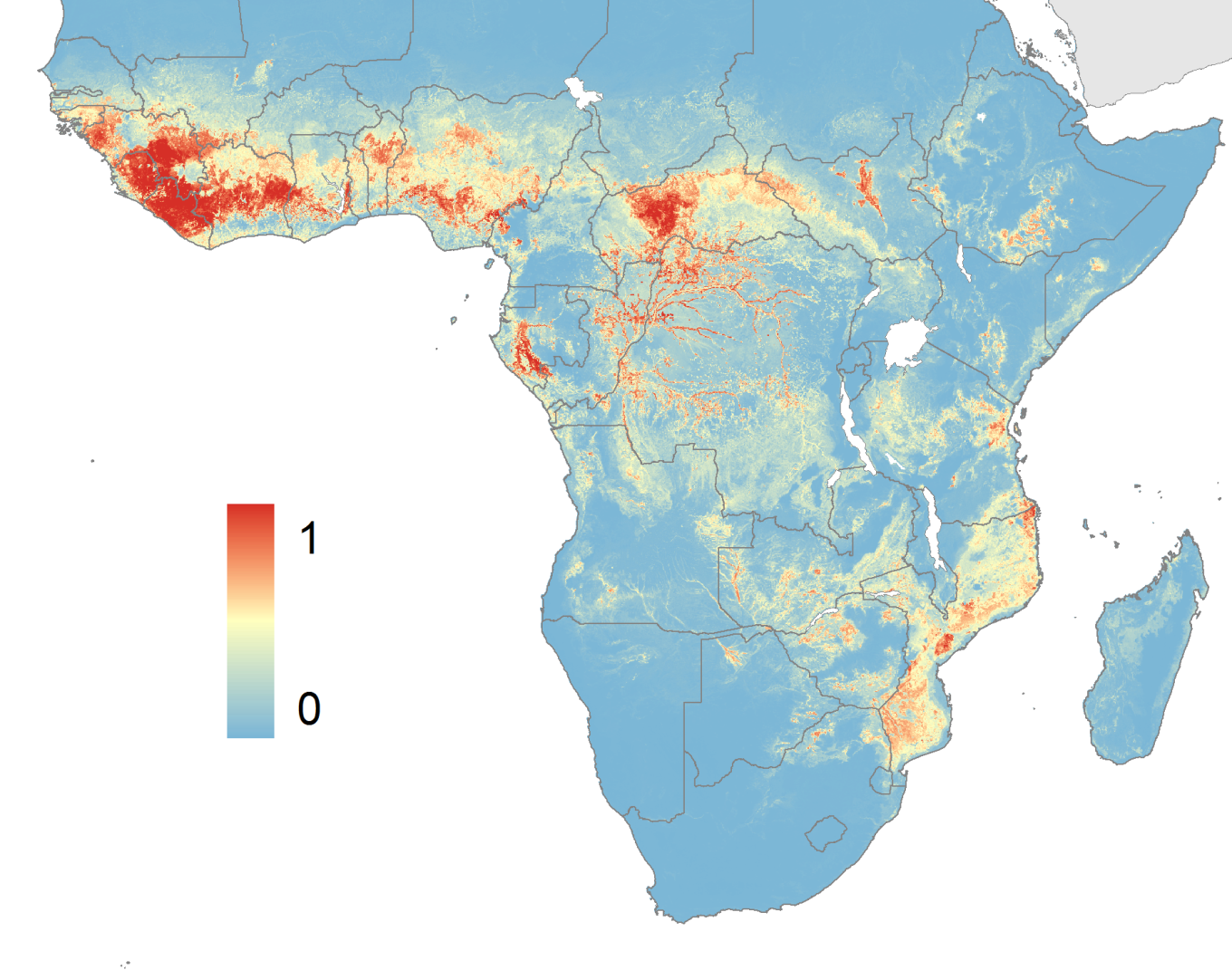


**Figure S2.3. Predicted geographical distribution of the zoonotic niche for Lassa virus using a 4:1 ratio diagnostic weighting schema** **for human or animal infections diagnosed via PCR/viral isolation and serological tests, respectively (Model 3).**

The scale reflects the environmental suitability for zoonotic transmission of Lassa virus. Areas closer to 1 (red) are more suitable than those closer to 0 (blue). The area under the curve statistic, calculated under a stringent cross-validation procedure is 0.78±0.02.


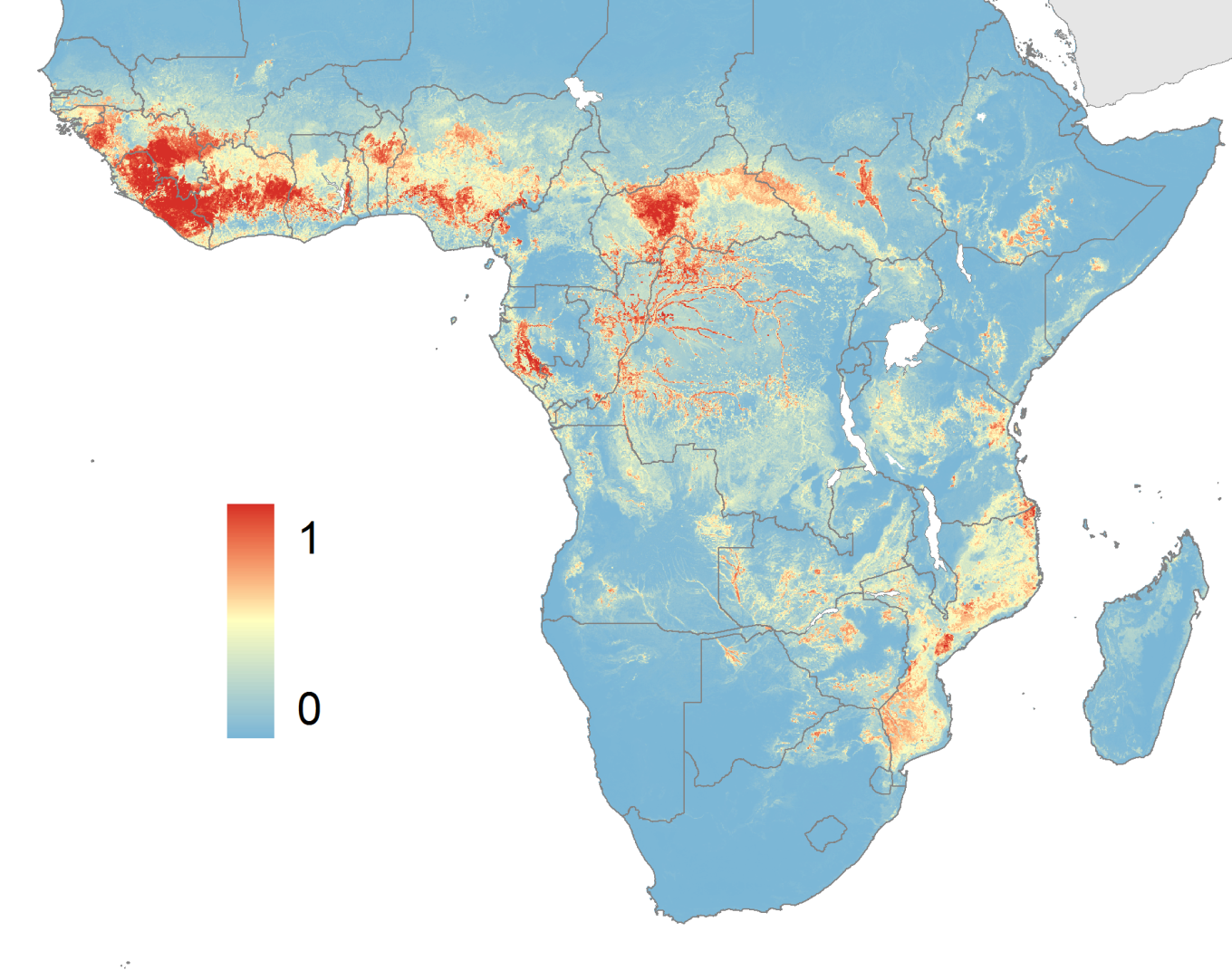


**Figure S2.4. Predicted geographical distribution of the zoonotic niche for Lassa virus for human or animal infections diagnosed via PCR/viral isolation tests (Model 4).**

The scale reflects the environmental suitability for zoonotic transmission of Lassa virus. Areas closer to 1 (red) are more suitable than those closer to 0 (blue). The area under the curve statistic, calculated under a stringent cross-validation procedure is 0.73±0.04.


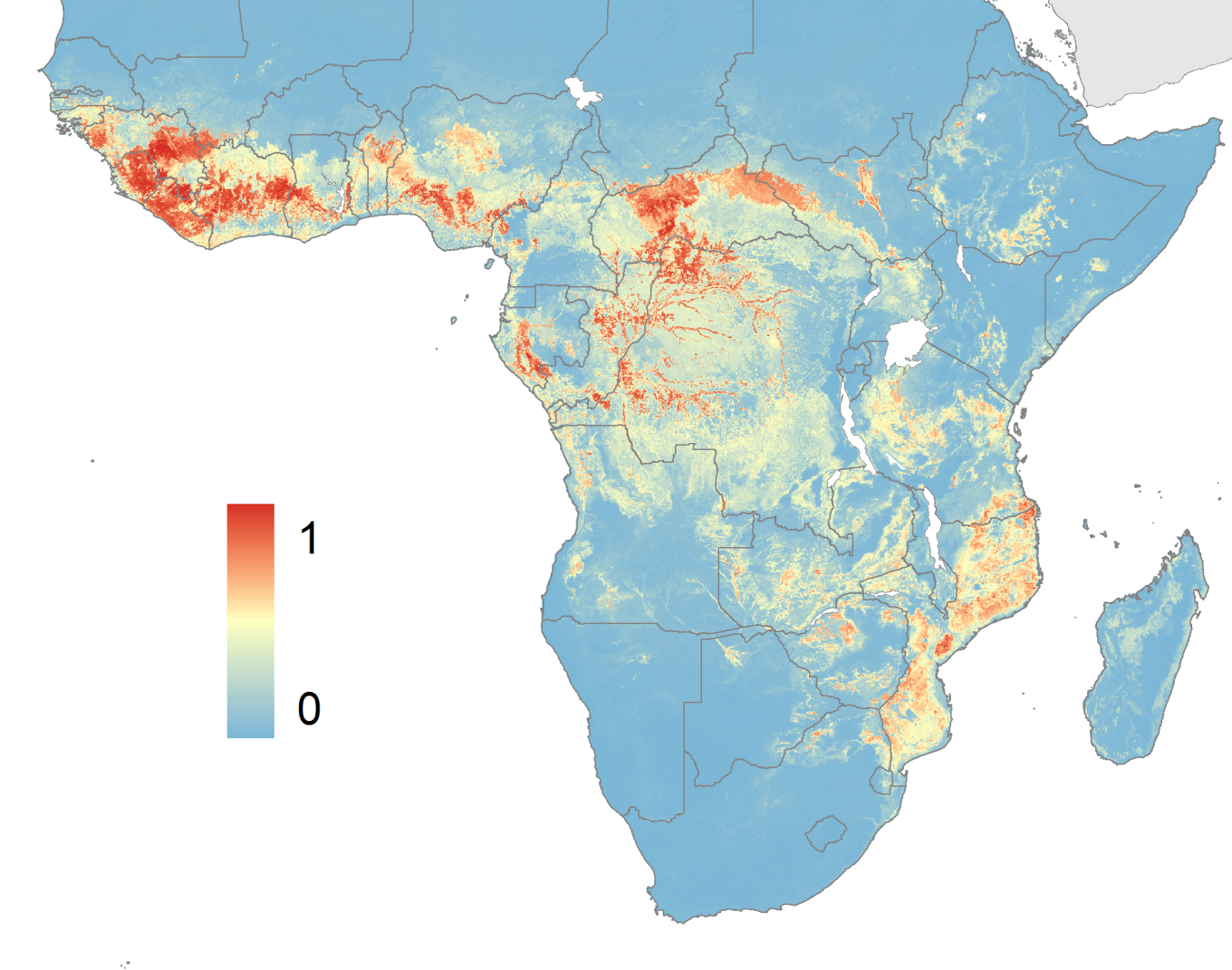


**Figure S2.5. Predicted geographical distribution of the zoonotic niche for Lassa virus using a 1:1 ratio diagnostic weighting schema for human infections diagnosed via PCR/viral isolation and serological tests, respectively (Model 5).**

The scale reflects the environmental suitability for zoonotic transmission of Lassa virus. Areas closer to 1 (red) are more suitable than those closer to 0 (blue). The area under the curve statistic, calculated under a stringent cross-validation procedure is 0.77±0.02.


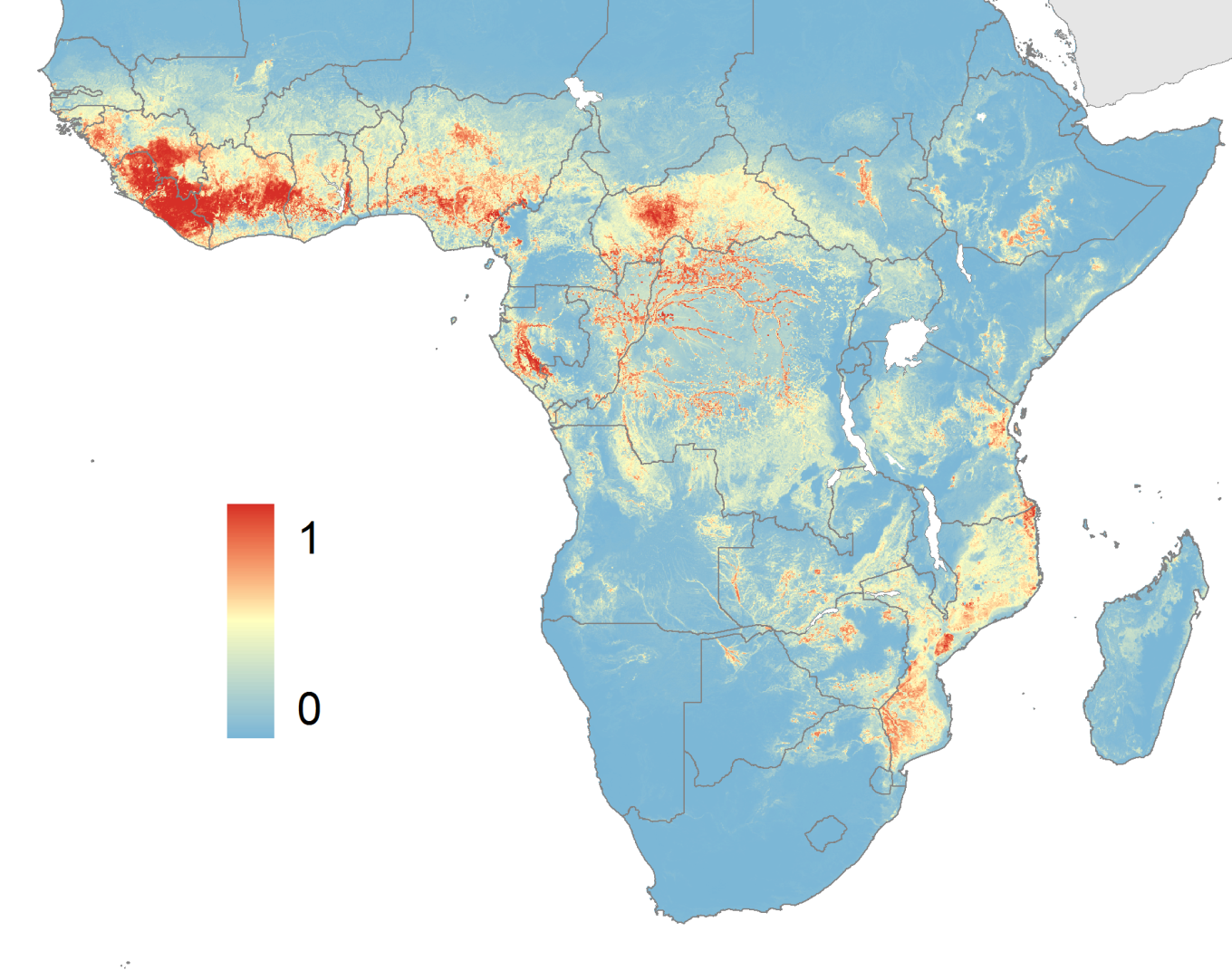


**Figure S2.6. Model 1 prediction range.**

The difference between the 5% and 95% confidence interval of predicted values was calculated. Areas in red have the greatest range in prediction values whilst areas in white, the smallest. The maximum range of pixel values is 0.800275.


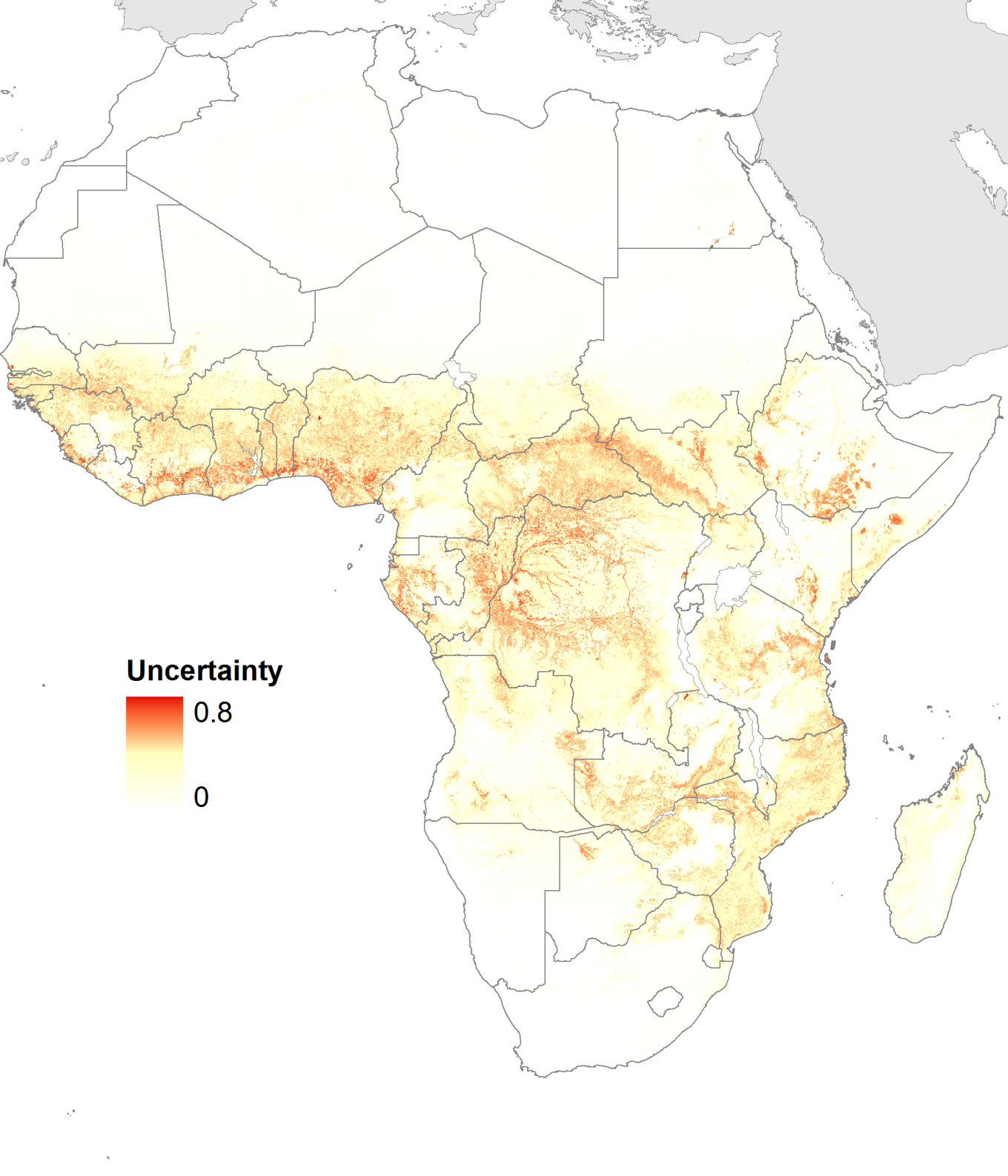


**Table S2.1. Summary statistics for model outputs. Relative contributions for each of the top five predictors are reported as a percentage**

| **Statistic** | **Model 1** | **Model 2** | **Model 3** | **Model 4** | **Model 5** |
| --- | --- | --- | --- | --- | --- |
| AUC ± s.d | 0.79±0.02 | 0.79±0.02 | 0.78±0.02 | 0.73±0.04 | 0.77±0.02 |
| 1^st^ predictor | Mean EVI:  26.5% | Mean EVI:  25.6% | Mean EVI:  25.0% | Mean EVI:  24.5% | Night-time mean LST: 25.3% |
| 2^nd^ predictor | Night-time mean LST: 19.2% | Night-time mean LST: 17.9% | Night-time mean LST: 17.2% | Night-time mean LST: 18.1% | Day-time mean LST: 19.5% |
| 3^rd^ predictor | Predicted host distribution:  13.6% | Elevation (DEM):  14.3% | Elevation (DEM):  14.7% | Day-time mean LST: 15.8% | Mean EVI:  15.1% |
| 4^th^ predictor | Elevation (DEM):  11.7% | Predicted host distribution:  12.1% | Predicted host distribution:  12.5% | Mean PET:  12.2% | Predicted host distribution:  11.7% |
| 5^th^ predictor | Mean PET:  10.6% | Mean PET:  11.0% | Day-time mean LST: 11.3% | Elevation (DEM):  10.6% | Elevation (DEM):  9.9% |

AUC: area under the curve; DEM: digital elevations models; EVI: enhanced vegetation index; Host: the Natal multimammate mouse, *Mastomys natalensis*; LST: land surface temperature; PET: potential evapotranspiration.
