# Supplementary material for: Mapping the zoonotic niche of Lassa fever in Africa
Source: Trans R Soc Trop Med Hyg. 2015 Jun 17;109(8):483–92. doi: 10.1093/trstmh/trv047 (PMC4501400; doi:10.1093/trstmh/trv047)
Supplement: Supplementary Data [file supp_trv047_trv047supp_supplementaryinformation3.docx]

**Supplementary information S3: Partial dependency plots**

**Figure S3.1. Partial dependency plots for the predicted geographical distribution of the Natal multimammate mouse, *Mastomys natalensis***

***
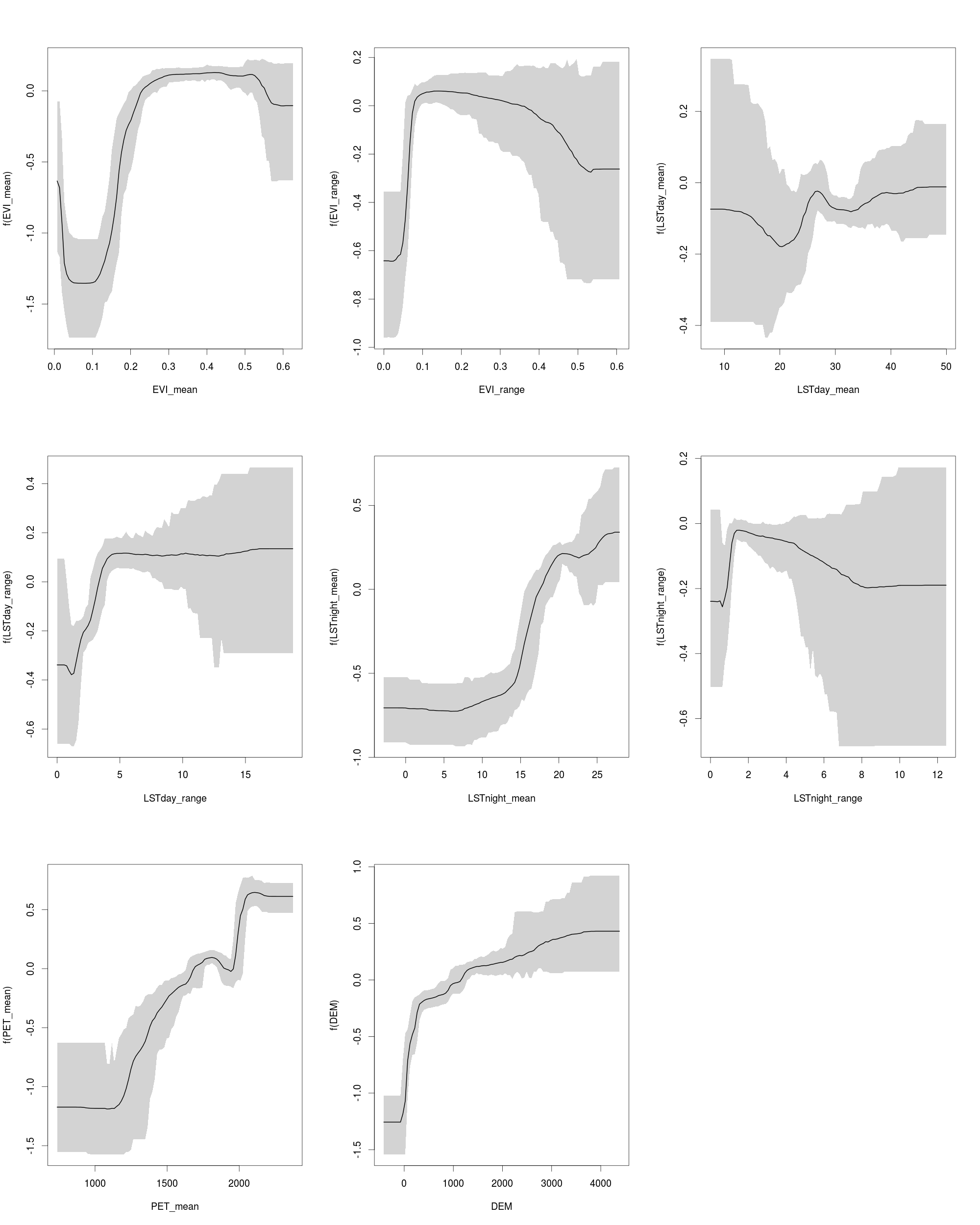
***

DEM: digital elevations models; EVI_mean: enhanced vegetation index mean; EVI_range: enhanced vegetation index range; LSTday_mean: day time land surface temperature mean; LSTday_range: day time land surface temperature range; LSTnight_mean: night time land surface temperature mean; LSTnight_range: night time land surface temperature range; PET_mean: potential evapotranspiration mean.

**Figure S3.2. Partial dependency plots for the predicted geographical distribution of the zoonotic niche for Lassa virus**

**
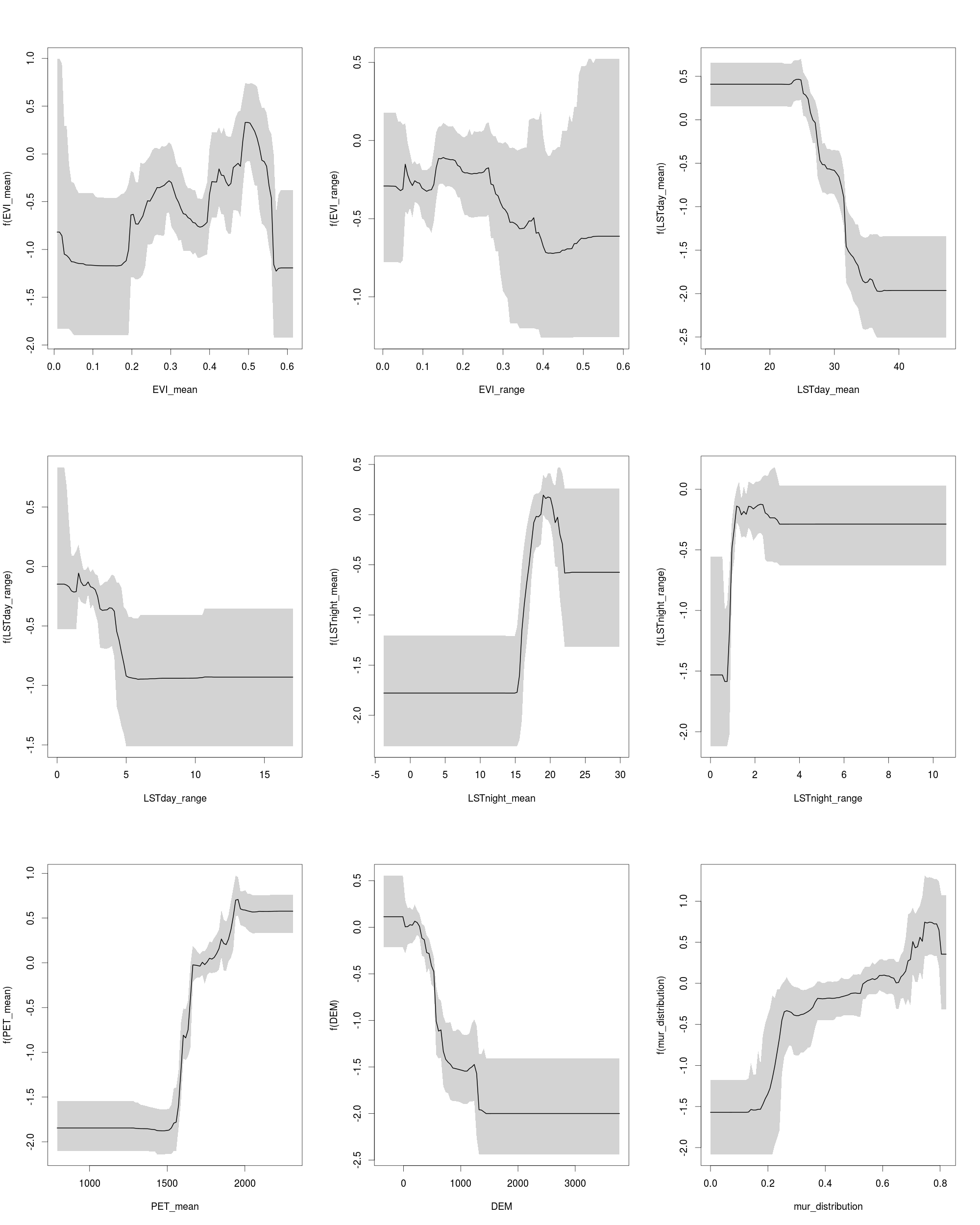
**

DEM: digital elevations models; EVI_mean: enhanced vegetation index mean; EVI_range: enhanced vegetation index range; LSTday_mean: day time land surface temperature mean; LSTday_range: day time land surface temperature range; LSTnight_mean: night time land surface temperature mean; LSTnight_range: night time land surface temperature range; mur_distribution: predicted geographical distribution of the Natal multimammate mouse, *Mastomys natalensis;* PET_mean: potential evapotranspiration mean.
