# Supplementary material for: Mapping the zoonotic niche of Lassa fever in Africa
Source: Trans R Soc Trop Med Hyg. 2015 Jun 17;109(8):483–92. doi: 10.1093/trstmh/trv047 (PMC4501400; doi:10.1093/trstmh/trv047)
Supplement: Supplementary Data [file supp_trv047_trv047supp_supplementaryinformation4.docx]

**Supplementary information S4: population at risk**

**Figure S4.1. Predicted geographical distribution of at-risk populations to Lassa virus.**

The continuous environmental suitability surface was converted into a binary at-risk (red), not-at-risk (blue) pixel layer. The threshold probability for a 5 km x 5 km at-risk pixel was calculated to be equal to or greater than 0.6459206. Countries with borders outlined by a solid line are those where cases of Lassa fever have previously been reported. Countries with borders outlined by a dash line have not previously reported Lassa fever cases.


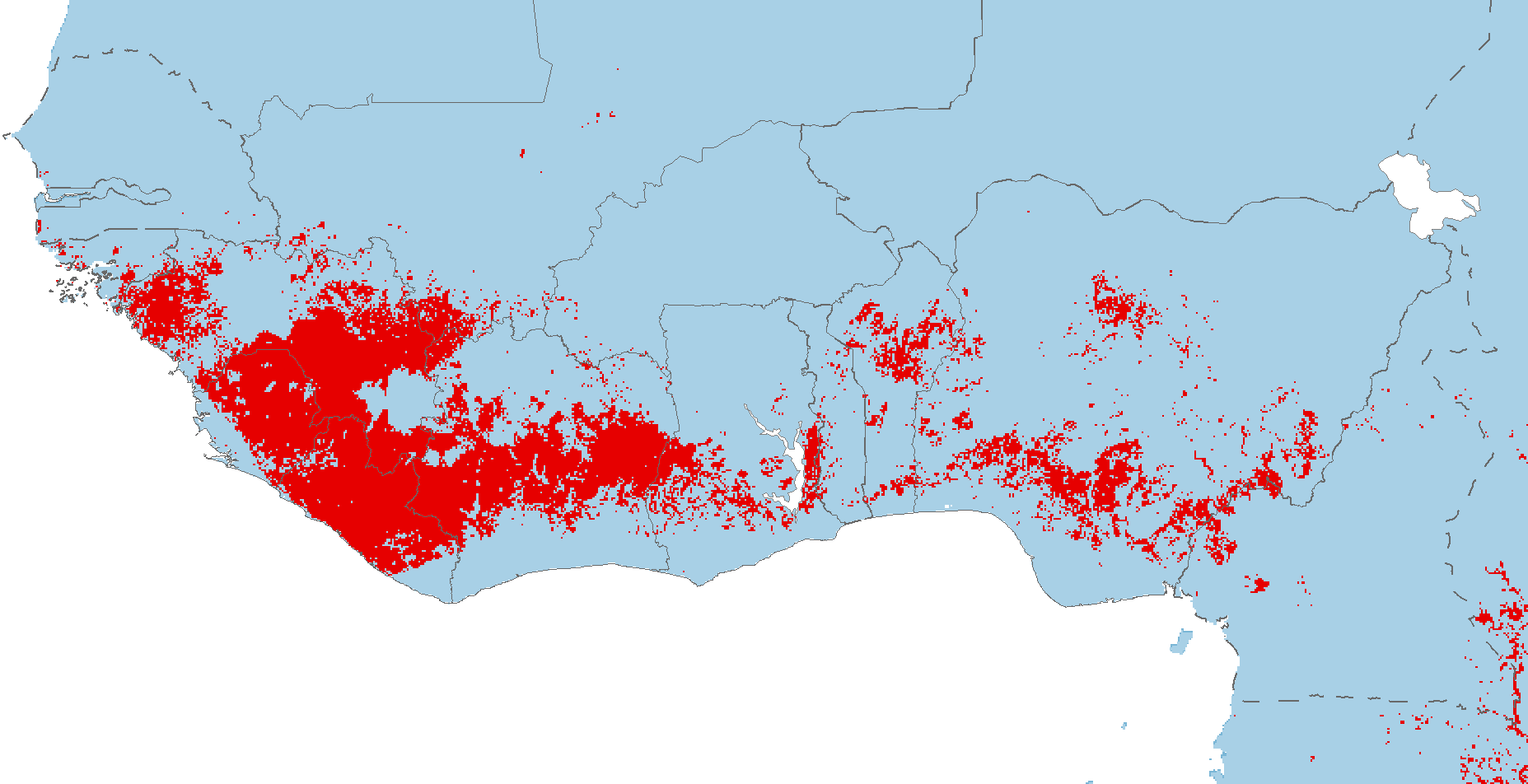


**Table S4.1. Location and size of at-risk populations to Lassa virus in countries with past Lassa fever cases**

| **Country** | **Population size** |
| --- | --- |
| Benin | 964 696 |
| Burkina Faso | 22 154 |
| Côte d'Ivoire | 6 885 680 |
| Ghana | 3 797 054 |
| Guinea | 4 930 742 |
| Liberia | 2 899 680 |
| Mali | 310 155 |
| Nigeria | 13 684 312 |
| Sierra Leone | 3 423 218 |
| Total | 36 917 691 |

**Table S4.2. Location and size of at-risk populations to Lassa virus in countries with no past Lassa fever cases**

| **Country** | **Population size** |
| --- | --- |
| Cameroon | 340 758 |
| Guinea-Bissau | 85 662 |
| Niger | 1687 |
| Senegal | 23 169 |
| Togo | 336 291 |
| Total | 787 567 |
